# Clinical guidelines in pediatric headache: evaluation of quality using the AGREE II instrument

**DOI:** 10.1186/1129-2377-15-57

**Published:** 2014-09-01

**Authors:** Pasquale Parisi, Nicola Vanacore, Vincenzo Belcastro, Marco Carotenuto, Ennio Del Giudice, Rosanna Mariani, Laura Papetti, Piero Pavone, Salvatore Savasta, Pasquale Striano, Irene Toldo, Elisabetta Tozzi, Alberto Verrotti, Umberto Raucci

**Affiliations:** 1 NESMOS Department, Chair of Paediatrics, Pediatric Headache Centre, Paediatric Sleep, Centre & Child Neurology, Faculty of Medicine & Psychology, “Sapienza University” c/o Sant’Andrea Hospital, Via di Grottarossa, 1035-1039 Rome, Italy; 2 National Centre for Epidemiology, Surveillance, and Health Promotion, National Institute of Health, Rome, Italy; 3 Neurology Unit, S. Anna Hospital, Como, Italy; 4 Department of Mental Health, Physical and Preventive Medicine, Center for Childhood Headache, Second University of Naples, Naples, Italy; 5 Department of Translational Medical Sciences, Section of Pediatrics, University of Naples Federico II, Naples, Italy; 6 Department of Neuroscience, Headache Centre, Bambino Gesù Children’s Hospital, IRCCS, Rome, Italy; 7 Department of Pediatrics, Child Neurology, Sapienza University of Rome, Rome, Italy; 8 Unit of Pediatrics and Pediatrics Emergency, University Hospital “Vittorio Emanuele”, Catania, Italy; 9 Department of Pediatrics, IRCCS Policlinico San Matteo Foundation, University of Pavia, Pavia, Italy; 10 Department of Neurosciences, Pediatric Neurology and Muscular Diseases Unit, Rehabilitation, Ophthalmology, Genetics, Maternal and Child Health, University of Genoa, “G. Gaslini” Institute, Genova, Italy; 11 Department of Woman and Child Health, Juvenile Headache Centre, University of Padua, Padua, Italy; 12 Child and Maternal Department, Headache centre University and S. Salvatore Hospital L'Aquila, L'Aquila, Italy; 13 Department of Pediatrics, University of Perugia, Perugia, Italy; 14 Paediatric Emergency Department, Bambino Gesù Children’s Hospital, IRCCS, Rome, Italy

**Keywords:** Guidelines, Pediatric headache, Agree II instrument, Quality of guidelines, Children

## Abstract

**Background:**

The Appraisal of Guidelines for Research and Evaluation (AGREE II) tool is a validated questionnaire used to assess the methodological quality of clinical guidelines (CGs). We used the AGREE II tool to assess the development process, the methodological quality, and the quality of reporting of available pediatric CGs for the management of headache in children. We also studied the variability in responses related to the characteristics of eleven Italian neuropediatric centers, showing similarities and differences in the main recommendations reported in CGs.

**Methods:**

A systematic literature search was conducted from January 2002 to June 2013 on Mediline, the Cochrane database, the National Guideline Clearinghouse website and the NHS evidence search tool, using the following terms: headache, cephalalgia, guidelines and children (MESH or text words). Six CGs providing information on the diagnosis and management of headache and specific recommendations for children were selected. Eleven neuropediatric centers assessed the overall quality and the appropriateness of all available CGs using of the AGREE II instrument.

**Results:**

Six CGs meeting the inclusion and exclusion criteria were identified and assessed by 11 reviewers. Our study showed that the NICE CGs was “strongly recommended” while the French and Danish CGs were mainly “not recommended”. The comparison between the overall quality score of the French CGs and the NICE CGs was statistically significant (6.54 ± 0.69 vs 4.18 ± 1.08; p =0.001). The correlation analysis between quality domain score and guideline publication date showed a statistically significant association only for the “editorial independence” domain (r = 0.842 p = 0.035). The intra-class coefficients showed that the 11 reviewers had the highest agreement for the Lewis CGs (r = 0.857), and the lowest one for the NICE CGs (r = 0.656). Statistical analyses showed that professionals from outpatient services dedicated pediatric headache assigned a higher overall quality score to the NICE CGs as compared to professionals from non-outpatient services (6.86 ± 0.38 vs 6.0 ± 0.82; p = 0.038).

**Conclusions:**

CGs resulted definitely of low-moderate quality and non “homogeneous”. Further major efforts are needed to update the existing CGs according to the principles of evidence based medicine.

## Background

About ten years ago, the World Health Organization (WHO) acknowledging the relevance of the issue, launched a global campaign to reduce the burden of headache, in collaboration with three major international headache non-governmental organization [1].

Headache is very frequently reported among children, even more frequently than among adults. It can have a strong impact on school performance [2], being the major cause of absence from school [3], and widely affecting other daily activities [4].

The individual and societal costs of headache disorders in children and adolescents are due to the high incidence, prevalence and lifetime prevalence of these conditions. In particular, the lifetime prevalence of headache disorders ranges from 70% to 80% in children of 13–15 years of age [5,6], while the lifetime prevalence of headache, considering all decades and according to both age- and gender-dependent variables, ranges between 12% and 18% as reported in international community-based studies [7].

A recent review of 64 cross-sectional studies reports an overall mean prevalence of headache of 54.4% and an overall mean prevalence of migraine of 9.1%. The mean prevalence of headache reported for girls was 59.2%, while for boys was 49.3%, while the mean prevalence of migraine was 10.5% for girls and 7.6% for boys [8].

Headache affects 3% to 8% of children aged ≥3 years, 19.5% of children aged 5, and 37% to 51.5% of children aged 7 [9,10], with an higher frequency in males before puberty, and in females after puberty [11].

A study based on parental reports may be an unreliable source of information on the frequency of headache in young children. In fact, it has been suggested that almost 36% of parents are unaware of their children suffering from headache [12]. Moreover, the increased incidence observed over the last 30 years is probably due to significant changes in children’s lifestyle [13].

Given the its high prevalence and the high degree of disability it can cause, it is no surprise that headache has become a relevant public health issue. Managing this condition, in fact, has its direct and indirect costs for the National Health Care System (NHCS), which are considerable and not easy to quantify, as children are not directly involved in the production cycle [14].

Agreement among independent physicians in the diagnosis of headache in children is often surprisingly poor and not adequately investigated [15]. Both the second [16] and the recently published third version [17] of the International Classification Headache Disorders (ICHD-III), stressed the need of more specific diagnostic criteria to increase the sensitivity, specificity and positive predictive value of the diagnosis of headache in children, which should be based not only on signs and symptoms, but also on the “behavior” and on some additional and peculiar diagnostic features (such as, moving into a dark room, squinting, turning off lights or photophobia, pallor, abdominal pain and many other autonomic signs and symptoms, increased sensitivity to odors or osmophobia) [18].

A further issue to be faced is the diagnosis and treatment of headaches in Pediatric Emergency Department. Children diagnosed and treated by healthcare professionals who are not expert in pediatric neurology, might risk to undergo inappropriate, unnecessary and harmful neuroradiological investigations.

Therefore, physicians facing this complex and multifaceted issues need appropriate CGs for the assessment, diagnosis and treatment of headaches in children.

The Appraisal of Guidelines for Research and Evaluation (AGREE II) assessment tool is a validated questionnaire used to assess the methodological quality of clinical guidelines (CGs) [19]. It has also been adopted by the World Health Organization (WHO) for the assessment of CGs [20].

This study aimed at using the AGREE II tool to assess the guideline development process, the quality of reporting of available pediatric CGs for the management of headache. The secondary endpoint was to assess the variability of provided answers in relation to the characteristics of each Italian SINP (Società Italiana di Neurologia Pediatrica) center enrolled in the study. A last objective was to identify similarities and differences in the main recommendations reported in the included CGs.

## Methods

Available CGs published from January 2002 to June 2013 were searched in Medline, the Cochrane, database, the National Guideline Clearinghouse website, and through the NHS evidence search service from using the following terms: “headache” or “cephalalgia”, and “practice guideline type” and “children” (MESH or text words). Further searches were carried out on the web sites of the main agencies that produce CGs, such as NICE (http://www.nice.org.uk), SIGN (http://www.sign.ac.uk), and SNLG (http://www.snlg-iss.it), and on the websites of scientific societies specialized in headache, such as the Italian Society for the Study of Headaches (http://www.sisc.it).

We included all CGs on the diagnosis and management of headache that included specific recommendations on infants and children. Guidelines referring exclusively to adults were excluded, and so were studies referring to previous publication.

The AGREE II tool is an international validated instrument for the assessment of the methodological quality of the CGs development process. The tool consists of 23 items organized in 6 domains: scope and purpose (3 items), stakeholder involvement (3 items), rigor of development (8 items), clarity and presentation (3 items), applicability (4 items) and editorial independence (2 items). Each item is scored with a 7-point Likert scale.

Scores range from 7 (strongly agree) to 1 (strongly disagree). The domain score is expressed as a percentage of the maximum possible score for that domain and is obtained by summing the scores of individual items.

One last item provides an overall judgment on the quality of the CGs, with a score ranging from 7 (higher possible quality) to 1 (lower possible quality).

A further 3-point scale (1 = not recommended- NR; 2 = recommended with provisos or modifications- R, and 3 = strongly recommended- SR) is included in the tool, which provides an overall judgment on whether the CGs should be recommended for use. Even though a threshold score is not defined, a domain score < 50% is usually considered of limited use.

All the 11 Italian centers specialized in the diagnosis and management of headache in children that refer to the SINP (Società Italiana di Neurologia Pediatrica), were included in the study. Eleven referents from all the included centers assessed the 6 CGs using the AGREE II instrument. Moreover, a specific questionnaire was administered to all referents to collect the following information: age, sex, years of specialization, years of clinical practice in the field of pediatric headache, presence in the center of an outpatient service for children with headache, number of first visits in 2012, number of control visits in 2012, number of patients visited in 2012, presence in the center of an emergency unit, estimate of the number of patients administered pharmacological treatments and non-pharmacological treatments, and estimate of the number of patients administered both treatments. The answers provided by the 11 references to this questionnaire were associated with those provided by the same referents to the AGREE II tool.

Statistical analyses were performed using t-tests for continuous variables and chi square tests for categorical variables. A correlation analysis between the answers provided to the AGREE II tool and specific variables collected through the additional questionnaire was also performed using the Pearson’s index. The intraclass coefficient correlation was used as measure of agreement between the 11 reviewers.

Data were analyzed with SPSS (version 21.0). A p value ≤ of 0.05 was considered as significant.

## Results

A total of six CGs were identified through Medline and the web sites of the main international guideline agencies and scientific societies [21-26].

Table 1 shows the AGREE II domain scores of the 6 GCs as assessed by the 11 reviewers. The NICE CGs [26] was judged as “strongly recommended” by 8 reviewers and “recommended with modifications” by 3 reviewers, while the French CGs [24] was “not recommended” by 5 reviewers, “recommended with modifications” by 5 reviewer and “strongly recommended” by 1 reviewer (Table 1). The difference between the overall quality scores of the NICE CGs and the French CGs was statistically significant (6.54 ± 0.69 vs 4.18 ± 1.08; p =0.001).

**Table 1:** AGREE II domain scores of the included CGs on the management of headache in children

**Guideline reference**	**Year**	**Diagnosis and/or treatment**	**Population**	**Domain 1**	**Domain 2**	**Domain 3**	**Domain 4**	**Domain 5**	**Domain 6**	**Overall quality score (range 1-7) (M ± SD)**	Overall assessment usage recommendations (n = 11)*
				***Scope and purpose %***	***Stakeholder involvement %***	***Rigor of development %***	***Clarity and presentation %***	***Applicability %***	***Editorial indipendence %***		
Lewis [21]	2002	Diagnosis	Children and adolescents	82.3	53	72.9	82.8	48.9	53	5.45 ± 1.13	SR = 8 (72.7%); R = 2 (18.2%) NR = 1 (9.1%)
SISC [22]	2003	Diagnosis and treatment	Children and adolescents	67.2	81.3	57.8	64.1	43.6	41.7	4.73 ± 1.49	SR = 3 (27.3%); R =5 (45.4%) NR = 3 (27.3%)
Lewis [23]	2004	Treatment	Children and adolescents	79.8	49	68.4	77.3	44.7	61.4	5.36 ± 0.81	SR = 5 (45.4%); R = 6 (54.6%)
Geraud [24]	2004	Diagnosis and treatment	Adult and children	60.6	55	42.8	67.2	31.4	56.8	4.18 ± 1.08	SR = 1 (9.1%); R = 5 (45.4%) NR = 5 (45.4%)
Bendtsen [25]	2012	Diagnosis and treatment	Adult and children	62.6	56.1	37.5	65.7	36	68.9	4.18 ± 1.54	SR =5 (45.4%); R =1 (9.1%) NR = 5 (45.4%)
NICE [26]	2012	Diagnosis and treatment	Adult and young people	92.9	86.4	90.7	94.9	75.4	74.2	6.54 ± 0.69	SR = 8 (72.7%); R = 3 (27.3%)

*AGREE* = Appraisal of Guidelines for Research and Evaluation; *CGs* = Clinical Guidelines.

*SR* = Strongly recommended; *R* = Recommended with modification; *NR* = Not recommended.

*number of pediatrics included in the study.

The “scope and purpose” domain is designed to assess the overall aim, the clinical questions covered by the CGs, and the patients to whom the CGs is meant to apply. The median score for this domain was 73.5% (range 60.6%-92.9%) with none of the included CG scoring < 50%.

The “stakeholder involvement” domain is meant to assess the degree of involvement in the development process of subjects from all the relevant professional groups, whether patients’ views and preferences have been sought, and the definition of the target users of the CGs. The median score for this domain was 55.5% (range 49%-86.4%) with only one CG scoring < 50% [23].

The “rigor of development” domain is aimed at assessing the methods used to search and select evidence, and to define the recommendations, and whether the health benefits, side effects, and risks have been considered. This domain also assesses the explicit link between the recommendations and the supporting evidence, whether the CGs has been externally reviewed by experts prior to its publication, and if a specific procedure for updating the CGs is provided. The median score for this domain was 63.1% (range 37.5% - 90.7%), with two CG scoring < 50% [24,25].

The “clarity and presentation” domain addresses the degree of clarity of the recommendations and the different options for the management of the target condition, and if the key recommendations are easily identifiable. The median score for this domain was 72.2% (range 64.1% - 94.9%) with none of the included CG scoring < 50%.

The “applicability” domain aims at assessing if the guideline is supported with tools for its application, and if the possible presence of organizational barriers to the implementation of the recommendations is discussed. This domain also assesses if the potential costs derived by the implementation of the recommendations are taken into account and if the CGs provide indications for the monitoring and/or audit process. The median score for this domain was 44.1% (range 31.4% - 75.4%) with five CG scoring < 50% [21-26].

**Figure 1:**
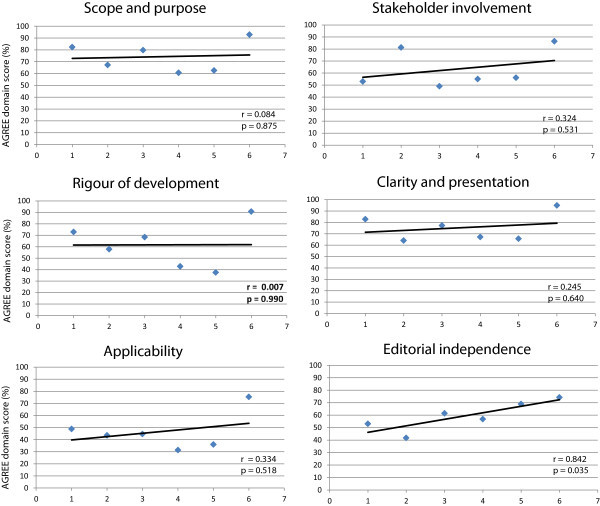
**Quality domain scores according to guideline publication date. 1**. Lewis [21]; **2**. SISC [22]; **3**. Lewis [23]; **4**. Geraud [24]; **5**. Bendtsen [25]; **6**. NICE [26]. For each numeric value of the caption matches a guideline.

The “editorial independence” domain is designed to assess the degree of independence from funding sources and the reporting of conflicts of interest by the CG development group. The median score for this domain was 59.1% (range 41.7% - 74.2%) with only one CG scoring < 50% [22].A correlation analysis between the quality domain scores and the CGs’ publication date showed a statistically significant association only for the “editorial independence” domain (r = 0.842 p = 0.035) (Figure 1).

The intraclass coefficients showed that the 11 reviewers reached the higher agreement when assessing the Lewis [21] CGs (r = 0.857), and the lower agreement when assessing the NICE CGs (r = 0.656) (Table 2). This is why the NICE CGs obtained the higher overall quality score, but with the lower agreement among reviewers (Tables 1 and 2).

**Table 2:** Assessment of the agreement among the 11 referents that assessed CGs with the AGREE II instrument

**Guideline reference**	**Year**	**Intraclass correlation coefficient**	**CI 95%**
Lewis [21]	2002	0.857	0.752 - 0.930
SISC [22]	2003	0.736	0.542 - 0.871
Lewis [23]	2004	0.804	0.660 - 0.904
Geraud [24]	2004	0.740	0.558 - 0.875
Bendtsen [25]	2012	0.745	0.548 - 0.873
NICE [26]	2012	0.656	0.404 - 0.832

Correlations between results from the assessment with the AGREE-II tool and the characteristics reported by the 11 reviewers were also investigated. The characteristics of the reviewers were collected through a specific questionnaire. Results from the questionnaire showed a wide heterogeneity in all the considered variables (Table 3). Statistical analyses showed that professionals from outpatient services specialized in pediatric headache assigned higher overall quality scores to the NICE CGs compared to those who do not work in similar structures (6.86 ± 0.38 vs 6.0 ± 0.82; p = 0.038). Reviewers who did not recommend the French CGs (n = 5) visited more patients in 2012 (a median value higher than 340) compared to those who recommended the CGs (R + NR = 6)(p = 0.036).

**Table 3:** Clinical profile of the 11 referents that assessed the included CGs with the AGREE II instrument and characteristics of their clinical centers

**Variables**	**M ± SD**	**Median**	**Range**
Age (yrs)	43.8 ± 11	39	32-64
Sex (M/F)	7/4	-	-
Years of specialization (yrs)	13.6 ± 11	9	0-33
Years of clinical practice in headache children (yrs)	13.6 ± 9.3	10	3-30
Is there a dedicated ambulatory service to children headache? (Y/N)	7/4	-	-
First ambulatory visits in 2012 year	238 ± 262	110	20-900
Control ambulatory visits in 2012 year	382 ± 421	250	40-1500
Total ambulatory visits in 2012 year	620 ± 681	340	60- 2400
Is there an emergency department? (Y/N)	9/2	-	-
Estimate of patients on pharmacological treatment (%)	58	60	40-80
Estimate of patients on non-pharmacological treatment (%)	28	25	5-50
Estimate of patients on both treatments (%)	26	20	10-75

Borderline significant correlations were observed between years of specialization in pediatrics of the referents and the overall scores assigned in the items of “editorial independence” domain (r = 0.535; p = 0.09), and between the overall quality scores assigned to all CGs and the number of control visits carried out by each participating center in 2012 (r = 0.503; p = 0.11). An inverse correlation was observed between years of clinical practice in pediatric headache of the referents and the overall scores assigned to the items in the “rigor of development” domain (r = - 0.526; p = 0.10).

Other statistical analyses carried out comparing the overall quality scores assigned to each CGs and the age (r = - 0.352), years of specialization (r = - 0.190), years of clinical practice in the field of children headache (r = - 0.376) and number of patients visited in 2012 (r = 0.487), but none of them was statistically significant.

## Discussion

In this study, we used the AGREE II questionnaire [19], a validated tool that had never been used before to assess the methodological quality of pediatric CGs, to assess the clinical practice CG development process, and the methodological quality and the quality of reporting of all available pediatric CGs for the management of headache.

Comparing available recommendations, the first data that clearly emerged (Table 1), was that the French and Danish CGs [24,25] scored both lower than the other available CGs, for the domain 3 (“Rigor of Development”: respectively, 42.8% and 37.5%) and domain 5 (“Applicability”: respectively, 31.4% and 36%). Five (45.4%) of the 11 referents from the SINP centers, according to these data, rated both CGs as “not recommended” (NR). These results are much likely due to the French and Danish CGs being focused on adults, with a minor section dedicated to the “diagnosis and treatment of headaches in developmental age”. This implies that both the French and Danish CGs, despite the lapse of eight years between their publication, are similarly unclear, with scarce information on children and adolescents, and are therefore poorly if not applicable. In fact, even though a threshold score is not defined, a domain score < 50% is usually considered as stated in the “methods” section, of limited use. Interestingly, the French CGs were “strongly recommended” (SR) by only 1 of the 11 referents (9.1%) and “recommended with modification” (R) by 5/11 referents (45.5%), while the Danish CGs received an exactly symmetrical score (respectively SR in 5/11 referents versus R in only 1 of them) (Table 1). These apparently conflicting data are balanced by those emerging from the “assessment of the agreement among the referents” reported in Table 2. This last statistical analysis of data is much more reliable than the overall assessment (Table 1), as it takes into account the “score” (Table 2) and not only an overall judgment on whether or not the CGs ought to be recommended (Table 1). In particular, Table 2 reports an interclass correlation coefficient of 0.740 for the French CG and of 0.745 for the Danish CGs, that is, two coefficients that can be considered practically overlapping (Table 2). The SR and R categories assigned in Table 2 to each CGs by the referents (see the legend for overall assessment usage recommendations) are much likely strongly affected by other important variables related to the characteristics of the center, as shown in Table 3.

Comparing the overall quality (Table 1), the NICE CGs (26) showed significantly higher scores versus the French and Danish CGs, thus CGs, being by far the best available CGs (6.54 ± 0.69 vs 4.18 ± 1.08; p =0.001) (Table 1). The NICE CGs showed also, the best scores for each domain and area (NICE CGs were “strongly recommended” by 8/11 reviewers and “recommended with modifications” by 3/11 reviewers, and “not recommended” by 0/11 reviewers).

However, some of the inconsistencies observed in the statistical analysis of results (as for the comparison between French and Danish CGs shown above) appeared to be even more evident for some aspects of the NICE CGs. A deeper analysis of NICE CGs, in fact, showed a lower intra-class correlation coefficient score (assessment of agreement between referents from 11 SINP Centers) (r = 0.656) (Table 2). This apparently strong inconsistency in the assessment of the NICE CGs among different SINP Centers can be explained by a different experience of the referents in the diagnosis and treatment of headaches in children. In particular, our analyses showed that some variables related to single SINP Centers (see the wide range of values reported in Table 3 for the variables “First, Control and Total, Ambulatory visits in 2012”, and “Years of the clinical practice”), can significantly vary according to the referents’ clinical profile, and to the characteristics of their clinical centers (Table 3). In other words, even though the 11 SINP centers included in this study are the most relevant and expert in the diagnosis and management of headache in children, referents from units such as pediatric neurology outpatient services with a higher number of first visits were able to give more appropriate and inconsistent answers in the assessment with AGREE II. Specifically, the answers provided by 11 reviewers (Table 3) showed a wide heterogeneity for all considered variables. Overall, professionals from outpatient services specialized in pediatric headache assigned higher overall quality scores to the NICE CGs compared to those who do not work in similar structures (6.86 ± 0.38 vs 6.0 ± 0.82; p = 0.038).

Furthermore, reviewers who did not recommend the French CGs (n = 5) visited more patients in 2012 (a median value higher than 340) compared to those that recommended the same guideline (R + NR = 6) (p = 0.036).

The “scope and purpose” domain is designed to assess the overall aim and the clinical questions covered by the considered CGs (Table 1), and the patients to whom the CG is meant to apply. None of the CGs included in this study scored < 50% (range 60.6%-92.9%). Only one of the included CGs (23) scored < 50% (range 49%-86.4%) in the “stakeholder involvement” domain (degree of involvement of individuals from all the relevant professional groups and patients’ views and preferences), while none of the included CGs scored < 50% in the “clarity and presentation” domain. Data from the “applicability domain” are remarkable, as only the NICE CGs [26] scored 75.4%, while all the other CGs (21–25) showed values < 50%. Correlation analyses between year of specialization in pediatrics and overall scores in the items of the “editorial independence” domain (r = 0.535; p = 0.09), and between the overall quality scores in the assessment of CGs and the number of control visits during 2012 (r = 0.503; p = 0.11) showed borderline significant results. An inverse correlation was observed between years of clinical practice in the field of pediatric headache and the overall scores assigned to the items of the “rigor of development” domain (r = - 0.526; p = 0.10).

One of the few positive aspects of the assessment is the “editorial independence” (the degree of independence from funding sources and the reporting of conflicts of interest by the CG development group ), which shows a progressive and significant increase from 2002 to 2012 (Figure 1), with a median score for the domain of 59.1% (range 41.7% - 74.2%) and only one CGs scoring < 50% [22]. Conflict of interest and editorial independence can be a relevant source of bias in the development of CGs, and high rates of conflict of interest among CGs authors have been reported in the past. The rates of disclosure conflict of interest by authors, and the availability to the public of this type of information are still unacceptably low, even though the rates of conflict of interest among guideline authors are currently decreasing [27]. A correlation analysis between quality domain score and guideline publication date, in particular, showed a statistically significant association for the “editorial independence” domain only (r = 0.842 p = 0.035) (Figure 1). This means that only one domain improves, from a qualitative point of view, in eleven years of CG production (Figure 1).How can this aspect be explained? Understanding why all other domains and areas of the AGREE II instrument did not significantly improved from 2002 to 2012, would probably be, in our opinion, much more useful than understanding the reason of the increase in the “editorial independence”, domain (Figure 1).

The “evidence based medicine” approach in the field of pediatric headache is unquestionably poor. Pediatricians admit that they would be more inclined to follow CGs if these were evidence-based, and therefore proved to improve outcomes [28]. Similarly, a study on Australian general practitioners showed that the most important factor leading the decision whether to follow a guideline or not, was its being evidence-based [29]. We believe that the reason for the lack of evidence on pediatric headache is to be found in the scarcity of funds devolved to research in pharmacological and non-pharmacological treatments for headaches in children . One of the reasons preventing either private or public investors from funding clinical studies in this field is the small expected difference between experimental drugs and placebo in children with headache, which means a huge sample is to be enrolled to see such a small effect. These issues are the reason why a number children, actually a small number due to the risk this practice implies, are prescribed drugs off label.

A last, but not less relevant issue is the heterogeneous and hyper-specialized education that characterizes this medical field. Specialists in “headache” can, in fact, come from specializations such as neurology, pediatrics, internal medicine, child neuropsychiatry. Addressing this issue is crucial to uniform the diagnosis, treatment and management of children with headache. The existence of this issue can also at least partially explain the conflicting results from the assessment carried out with the AGREE II tool. All CGs analyzed with the AGREE II tool in this paper refer to the criteria published in the International Classification of Headache Disorders (ICHD) for diagnostic purposes. Unfortunately, the first edition of he ICHD (ICHD-I) [30], which has been in use from 1988 to 2004, did not provide a clear distinction between diagnostic criteria for children and those for adults. This distinction was implemented in 2004 (ICHD II) [16] and maintained onwards this differentiation. The last edition of the ICHD, the “third” edition (ICHD III), has been published in July 2013 [17] because the issue of diagnosis is therefore relevant, as among the CGs included in the present paper (Table 1), only those published in 2012 [26] refer to diagnostic criteria specifically designed for children, while all other CGs (Table 1) published before the ICHD-II [16] refer to generic criteria.

Data on acute therapies for children with headaches are also few, but more consistent (acetaminophene, ibuprofene and triptans, for children older than 12 years), considering patients’ preferences, comorbidities and the risk of adverse events. Topiramate has recently [25,26] proved effective, safe and well tolerated for prophylactic purposes, while flunarizine, valproate and beta-blockers are already included in previously published CGs [22-24]. However, no drugs for the prevention of migraine in children are currently approved by both the Food & Drug Administration and European Medicine Agency, therefore, pediatricians are forced to prescribe drugs approved for adults or off-label (approved for clinical conditions other than migraine prevention).

To summarize an overall judgment of the existing CGs on the diagnosis and treatment of headaches in children, we can say, without any doubt, that currently available CGs are of low-moderate quality and non “homogeneous”. Huge gaps between literature-based evidence and best practice are not infrequent. This makes evident that the act of developing CGs is not enough, by itself, to change the everyday practice [31,32]. The existence of CGs does not change the practice if the reasons for changing are not evident to all professionals involved in the management of patients with a given condition. The success or failure of CGs is multi-factorial and is related to the characteristics of the development process and the implementation, to the provider and the patient beliefs and preferences, to the therapeutic setting, and to the limitations of methodological quality. However, one of the primary objective of CGs is its being be oriented toward the target population and/or setting in which it is meant to be applied (e.g. children), and only CGs oriented towards subjects of all age ranges could guarantee the highest compliance and could actually change clinical practice and eventually increase patients’ quality of life.

Additional RCTs and more controlled data are needed to help clinicians choose the most appropriate drugs for the treatment of this common clinical problem. New and innovative study designs are required to this purpose, to minimize the high placebo response observed in pediatric populations.

## Conclusion

In conclusion, this survey, which was carried out on behalf of the “Pediatric Headache Commission” of SINP, highlights that further research is strongly needed in this area, and that existing CGs should be updated basing on the principles of evidence based medicine.

## Competing interests

All authors declare that they have no competing interests.

## Authors’ contributions

PP (Pasquale Parisi) and UR: They participated to the conception and design of the study, the acquisition of data, the statistical analysis and interpretation of data; they also coordinated and drafted the first version of the manuscript, and critically revised its intellectual contents. Finally, they approved the submitted version of the manuscript. NV: He participated in the design of the study, in the statistical analysis and interpretation of data and critically revised the intellectual content of the final manuscript. VB, MC, EDG, RM, LP, PP (Piero Pavone), SS, PS, IT, ET, AV: They collected data from each of the eleven SINP Centres that accepted to participate in the study, filling the AGREE II questionnaire (according to the design of the study) and filling also the “form” collecting all data related to the “skills” and characteristics of the health care of the neuropaediatric outpatient service at each SINP Centre (see paragraph: Materials and Methods). They all also participated in critically revising the intellectual contents of the final manuscript. All authors read and approved the final manuscript.
